# The efficacy of local continuous chemotherapy and postural drainage in combination with one-stage posterior surgery for the treatment of lumbar spinal tuberculosis

**DOI:** 10.1186/s12891-016-0921-2

**Published:** 2016-02-09

**Authors:** Yongchun Zhou, Zongrang Song, Jing Luo, Jijun Liu, Yunfei Huang, Yibin Meng, Wentao Wang, Dingjun Hao

**Affiliations:** Department of Spine Surgery, Honghui Hospital, Xi’an Jiaotong University College of Medicine, Xi’an, People’s Republic of China

**Keywords:** Spinal tuberculosis, Bone grafting, Chemotherapy, Posterior debridement, Instrumentation

## Abstract

**Background:**

The objective of this study was to compare the outcomes of one-stage posterior surgery involving debridement, bone grafting, and instrumentation with and without local continuous chemotherapy and postural drainage for the treatment of lumbar spinal tuberculosis.

**Methods:**

From January 2009 to January 2013, 109 patients with lumbar spinal tuberculosis were treated in our center using a posterior surgical approach. Patients underwent one-stage posterior debridement, bone grafting, and instrumentation, without (group A) and with (group B) local continuous chemotherapy and postural drainage. Clinical and radiographic results for the two groups were analyzed and compared. Clinical efficacy was evaluated based on surgery duration and blood loss. The Frankel scale was used to evaluate neurological function. A visual analog scale was used to assess low back pain. Bone graft fusion and instrumentation failure were monitored by radiography, and tuberculosis activity was monitored by erythrocyte sedimentation rate (ESR) and C-reactive protein testing.

**Results:**

Groups A and B contained 52 and 57 patients, respectively. Patients were followed for 18–36 (mean, 26.64 ± 4.2) months. All bone grafts ultimately fused, but the fusion rate was significantly more rapid in group B [6.4 ± 0.5 (range, 5–10) months] than in group A [8.9 ± 0.6 (range, 6–12) months; *P* < 0.05]. At 6 weeks postoperatively, ESR levels differed significantly between groups A and B (24.6 ± 1.5 *vs*. 16.3 ± 1.1 mm/h; *P* < 0.05). ESR levels normalized in both groups at 16 weeks.

**Conclusions:**

Local continuous chemotherapy and postural drainage effectively eliminated infection foci caused by abscess remnants and accelerated interbody bone fusion in patients with lumbar spinal tuberculosis undergoing one-stage posterior surgery involving debridement, bone grafting, and instrumentation.

## Background

The incidence of spinal tuberculosis has increased in developing countries in recent years [1]. Tuberculosis can lead to kyphotic deformity of the spine associated with paraplegia. Spinal tuberculosis can be treated surgically, with the goals of achieving adequate decompression and debridement, reinforcing spinal stability, and correcting and preventing deformity. Approaches to this surgical treatment include anterior spinal fusion, anterior spinal fusion with posterior spinal fusion, posterior spinal fusion alone, and posterior spinal fusion followed by anterior spinal fusion [2, 3]; the best surgical approach, however remains a matter of debate.

Anterior debridement provides direct access to the infected focus and facilitates vertebral reconstruction. The disadvantages of the anterior-only approach include insufficient kyphosis correction and the potential for major loss of correction postoperatively. This approach also may involve division of the diaphragm, and is associated with a low rate of fusion and a high rate of vascular complications [4, 5]. The combined anterior and posterior surgical approach is accepted widely because it can overcome stability-related drawbacks. With the development of contemporary posterior segmental spinal instrumentation systems, one-stage posterior debridement/bone grafting/instrumentation is used increasingly to treat active spinal tuberculosis. Some surgeons have reported the performance of this one-stage surgery via the posterior approach alone [6–9].

Antituberculous chemotherapy is the mainstay of tuberculous spondylitis treatment. Few reports have described the treatment of tuberculous spondylitis with psoas abscess by percutaneous drainage and local continuous chemotherapy [10], and no report has described the use of a one-stage approach for this condition involving posterior debridement, bone grafting, local continuous chemotherapy, postural drainage, and instrumentation. Here, we report on the treatment of lumbar tuberculosis with psoas abscess using one-stage posterior debridement, interbody vertebral fusion, and posterior instrumentation, with and without local chemotherapy and postural drainage. We examined the effects of local continuous chemotherapy and postural drainage on posterior debridement, bone grafting, and instrumentation in this treatment approach.

## Methods

### Patient population

We enrolled adult patients undergoing surgical treatment of active lumbar spinal tuberculosis, with and without protrusion deformity, between January 2009 and January 2013 in this retrospective study. Diagnoses of lumbar tuberculosis were based on clinical symptoms, laboratory findings, and radiographic [X-ray, computed tomography (CT), and magnetic resonance imaging] evidence, and confirmed postoperatively by pathological examination. Additional inclusion criteria were: 1) low back pain accompanied by neurological symptoms, and 2) lesions involving one vertebral body or two adjacent vertebral bodies. Exclusion criteria were: 1) lesions involving more than two vertebral bodies; 2) flow injection abscess or fistula formation; 3) active pulmonary tuberculosis or spinal tuberculosis with no obvious relief of clinical symptoms [weight loss, low-grade fever, night sweats, fatigue, decline in erythrocyte sedimentation rate (ESR)] after 2 weeks of regular chemotherapy; 4) lack of nerve injury; and 5) cardiopulmonary dysfunction contraindicating surgery. The study protocol was approved by the ethics review committee of Honghui Hospital, Xi’an Jiaotong University College of Medicine, and all patients provided written informed consent to study participation and the use and publication of data for research purposes.

### Preoperative preparation

All patients were treated with HREZ chemotherapy (isoniazid, 300 mg/day; rifampicin, 450 mg/day; ethambutol, 750 mg/day; pyrazinamide, 1.5 g/day) for 2–4 weeks prior to surgery.

### Operative technique

**Fig. 1:**
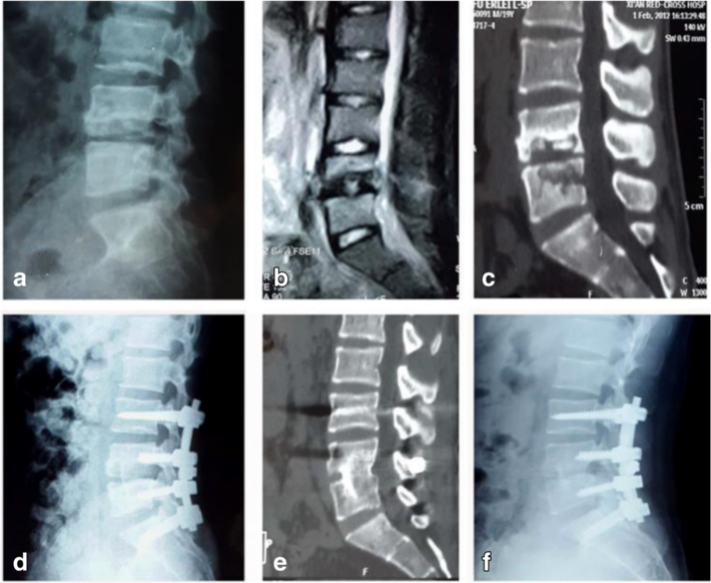
Images from a 21-year-old man with lumbar spinal tuberculosis who underwent one-stage posterior debridement, bone grafting, and instrumentation. **a**–**c** Preoperative X-ray, MR, and CT images showing the destruction of L4 and L5 and paravertebral abscess formation. **d** Postoperative lateral X-ray showing fixation of L3–S1. **e**, **f** At the 24-month follow-up examination, fixation and interbody vertebral fusion were satisfactory, with no sign of tuberculosis recurrence

**Fig. 2:**
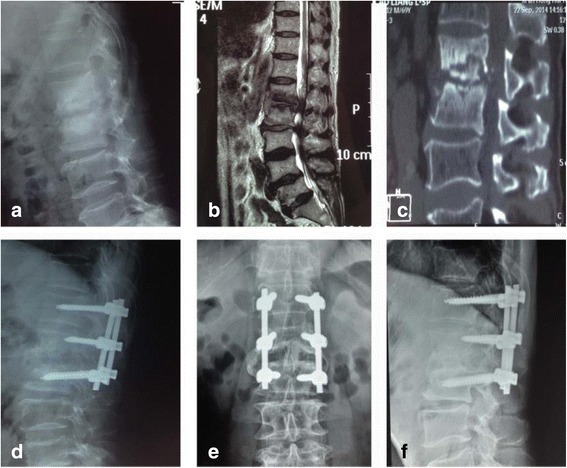
Images from a 70-year-old man with lumbar spinal tuberculosis who underwent one-stage posterior debridement, bone grafting, and instrumentation with local continuous chemotherapy and postural drainage. **a**–**c** Preoperative X-ray, MR, and CT images show the destruction of L1 and L2 and paravertebral abscess formation. **d** Postoperative lateral X-ray showing fixation of T12–L2. **e**, **f** X-rays obtained 6 months postoperatively show successful interbody bony fusion

Patients underwent one-stage posterior debridement, bone grafting, and instrumentation, alone (group A) or with local chemotherapy and postural drainage (group B). Under general endotracheal anesthesia, each patient was placed in the prone position. Through a midline incision, extraperiosteal dissection was performed to expose the posterior spinal elements, including the laminae, facets, and transverse processes, to one vertebra below and above the involved segments. Pedicle screws (Adena®; Sanyou Co., Shanghai, China) were placed in the two normal vertebral bodies nearest the lesion, with the number of screws determined by the extent of the lesion and vertebral body damage. After pedicle screws were implanted, a pre-bent temporary internal fixation rod (Adena®; Sanyou Co.) was placed on one side of the focus and stabilized to avoid spinal cord injury induced by instability of the spine during decompression and focal debridement. The vertebral lamina and superior and inferior articular processes on the affected side were drilled; the necrotic disc, collapsed vertebrae, and paravertebral abscess were removed; spinal pressure was relieved with a disposable infusion tube; and a suitable flush tube (SHSJ-50; Songhu Plastic Machinery Co., Ltd, Dongguan, China) was plunged into the psoas abscess. The abscess was then washed with hydrogen peroxide and saline until no pus outflow occurred. Suitable massive autologous bone grafts from the ilium were inserted in the bone trough to reconstruct the vertebrae and the screws were tightened to achieve internal fixation. Kyphosis was slowly rectified by compressing and stretching the internal fixation instrument (Figs. 1 and 2), If unilateral lesions were not eliminated, a second pre-bent temporary internal fixation rod was placed on the other side for stabilization and the first temporary fixed rod was removed. The same process was completed on the opposite side of the lesion. X-rays were obtained to confirm the appropriateness of bone graft position, intervertebral height recovery, and correction of kyphosis. Drainage tubes were placed in patients in group A. Postural drainage tubes were placed in patients in group B, and a suitable infusion tube was placed at the lesion site in each of these patients for daily irrigation with isoniazid (1.0 g in 500 ml saline). Drainage and incision sites were sutured. Resected specimens were submitted for bacterial culturing and pathological diagnosis.

### Postoperative care

Patients received antibiotics for 3 days postoperatively and routine postoperative enhanced nutritional support. Drainage tubes of patients in group A were removed after 72 h, except in the presence of an extensive paraspinal abscess; in such cases, tubes were left indwelling until drainage was <50 ml/24 h. Continuous chemotherapy and postural drainage were continued for 20–30 days for patients in group B, after which the tubes were removed.

Patients continued oral HREZ chemotherapy for 6 months postoperatively; pyrazinamide was then discontinued and patients received 9–12 months of HRE chemotherapy. After discharge, liver function was assessed monthly and the ESR was measured preoperatively and 6 and 16 weeks postoperatively; after 16 weeks, the ESR was measured every 2 months. Ambulation with a brace was allowed 6–8 weeks after surgery, as premature ambulation may lead to displacement of the bone graft. Patients used a brace for at least 3 months. The patients were allowed to perform non-weight-bearing daily activities 6–8 weeks after surgery and normal weight-bearing activities after interbody fusion had been confirmed radiographically (by X-ray or CT).

### Postoperative evaluation

Preoperatively and at the final follow up, the Frankel scale was used to evaluate neurological function. Preoperatively and 6 and 16 weeks postoperatively, the Oswestry Disability Index (ODI) was used to evaluate neurological function changes. The ODI is the sum of points on 10 items characterizing disability (0–5 points/item) [11]. A visual analog scale (VAS) was used to assess low back pain, and tuberculosis activity was monitored with ESR and C-reactive protein (CRP) tests. Bone graft fusion and instrumentation failure were monitored by radiography. Fusion status was evaluated according to Kim et al. [12]. When plain radiographs did not yield clear findings, CT was performed.

### Statistical analysis

All data are presented as means ± standard deviations (SDs). Frankel grade preoperatively and at the last follow up were compared using the Wilcoxon signed rank test. Student–Newman–Keuls tests were used to compare changes in various laboratory and physical parameters in the two groups. *P* < 0.05 was considered to be statistically significant. All analyses were conducted using SPSS 19.0 software (SPSS, Inc., Chicago, IL, USA).

## Results

Of the 109 patients enrolled in this study, 52 underwent one-stage surgery without local chemotherapy (group A) and 57 underwent this surgery with chemotherapy (group B). Group A comprised 30 men and 22 women with a mean age at the time of initial operation of 49.9 ± 15.9 (range, 20–71) years. Group B comprised 36 men and 21 women with a mean age of 50.5 ± 16.1 (range, 21–73) years. Patient, surgical, and postoperative characteristics are summarized in Table 1.

**Table 1:** Patient and surgical characteristics and outcomes of the two groups

Characteristic	Group A (*n* = 52)	Group B (*n* = 57)
Sex (male)	30 (57.7 %)	36 (63.2 %)
Age at initial operation (years)	49.9 ± 15.9 (20–71)	50.5 ± 16.1 (21–73)
Operation time (min)	215.5 ± 35.0 (146–276)	222.6 ± 39 (157–279)
Intraoperative bleeding (ml)	520.1 ± 59 (250–1000)	522.3 ± 54 (253–1000)
Time to abscess disappearance (postoperative months)^*^	7.4 ± 0.7 (6–12)	5.4 ± 0.6 (5–10)
Time to bone fusion (postoperative months)^*^	8.9 ± 0.6 (6–12)	6.4 ± 0.5 (5–10)

Data are presented as *n* (%) or mean ± standard deviation (range)

^*^ *P* < 0.05

Thirty-three patients in group A and 35 patients in group B had different degrees of major vertebral abscess near the psoas. The distributions of involved vertebral bodies and lumbar fusion sites are shown in Table 2. Preoperative ESRs were ≤40 mm/h and hemoglobin levels were ≥100 g/L. Mean operation time and amount of intraoperative bleeding did not differ between groups. Tuberculosis was confirmed by bacterial culture or pathology in all patients; cultures from 70 patients were positive for *Mycobacterium tuberculosis*.

**Table 2:** Distributions of involved vertebral bodies and lumbar fusion sites

Group	Affected vertebra(e)							Fusion site			
	L2	L2–3	L3	L4	L3–4	L4–5	L5	L2–3	L3–4	L4–5	L5–S1
A (*n* = 52)	2 (3.8 %)	6 (11.5 %)	5 (9.6 %)	6 (11.5 %)	9 (17.3 %)	19 (36.5 %)	5 (9.6 %)	8 (15.4 %)	13 (25 %)	25 (48.1 %)	6 (11.5 %)
B (*n* = 57)	3 (5.3 %)	7 (12.3 %)	7 (12.3 %)	15 (26.3 %)	7 (12.3 %)	12 (21.1 %)	6 (10.5 %)	6 (10.5 %)	13 (22.8 %)	27 (47.4 %)	11 (19.3 %)

Patients were followed postoperatively for 18–36 (mean, 26.64 ± 4.2) months. All incisions healed well, and no chronic sinus formation or tuberculosis recurrence was observed. A unilateral psoas abscess was observed in one patient in group A at 10 months postoperatively, but no abscess was observed at 12 months postoperatively. No patient in group B experienced operative complications. No severe neurological complication was observed in either group.

Abscesses disappeared completely within significantly less time in group B than in group A (*P* < 0.05; Table 1). Fusion time was significantly shorter in group B than in group A (*P* < 0.05; Table 1).

Preoperative and 6-week postoperative ESR and CRP levels differed significantly within groups A and B, respectively; 6-week and 16-week values also differed within groups (all *P* < 0.05; Table 3). ESR and CRP values differed significantly between groups at 6 weeks, but not at 16 weeks, postoperatively (*P* < 0.05).

**Table 3:** Measures of baseline severity and surgical outcomes of the two groups

Measure	Group A	Group B
ODI, %		
Preoperative	58.2 ± 14.1	54.2 ± 12.1
6 weeks postoperative	20.3 ± 4.2^*^	20.8 ± 3.9^*^
16 weeks postoperative	2.2 ± 0.4^**^	1.8 ± 0.3^**^
VAS		
Preoperative	6.1 ± 1.2	6.2 ± 1.1
6 weeks postoperative	2.5 ± 1.1^*^	2.4 ± 1.2^*^
16 weeks postoperative	0.7 ± 0.4^**^	0.7 ± 0.5^**^
CRP (mg/L)		
Preoperative	19.2 ± 5.1	18.7 ± 5.2
6 weeks postoperative^***^	9.0 ± 1.9^*^	7.1 ± 1.9^*^
16 weeks postoperative	3.1 ± 0.5^**^	2.7 ± 0.5^**^
ESR (mm/h)		
Preoperative	39.6 ± 9.9	39.1 ± 9.8
6 weeks postoperative^***^	24.6 ± 1.5^*^	16.3 ± 1.1^*^
16 weeks postoperative	10.1 ± 1.0^**^	9.3 ± 1.1^**^

*ODI* Oswestry Disability Index, *VAS* visual analog scale, *ESR* erythrocyte sedimentation rate, *CRP* C-reactive protein

^*^ *P* < 0.05 *vs*. preoperative

^**^ *P* < 0.05 *vs*. 6 weeks postoperative

^***^ *P* < 0.05, group A *vs*. group B

All patients demonstrated neurological deficits preoperatively (Frankel grades C and D; Table 4). At the final follow up, neurological parameters had returned to normal in 50 patients in group A and 54 patient in group B; long-term neurological compression prevented such recovery in two patients in group A and three patients in group B. No significant difference in neurological parameters was observed between groups at final follow up, but significant differences between preoperative and final follow-up results differed significantly in each group (both *P* < 0.05). At 6 and 16 weeks postoperatively, ODI and VAS values did not differ between groups. These values differed significantly from baseline at 6 weeks postoperatively in both groups (all *P* < 0.05).

**Table 4:** Neurological recovery according to Frankel grade

	Group A					Group B				
Time point	A	B	C	D	E	A	B	C	D	E
Preoperative			4	48				6	51	
Final follow-up^*^				2	50				3	54

^*^ *P* < 0.05 *vs*. preoperative

## Discussion

Since the first description of tuberculosis in 1877, various methods of surgical management in patients with lumbar tuberculous spondylitis have been reported [13, 14]. Recent technological advances have enabled one-stage surgical treatment involving posterior debridement, bone grafting, and instrumentation. One-stage surgery with posterior transpedicular debridement and instrumented fusion, but without anterior debridement, has been shown to achieve satisfactory stabilization and kyphosis prevention in patients with spinal tuberculosis [6–9, 15]. Guven et al. [16] also reported good results of isolated posterior instrumentation and fusion without anterior debridement. Li et al. [7] reported that single-stage posterior surgery with instrumentation caused less operative trauma and was a good method for the treatment of lumbar spinal tuberculosis.

This study examined the outcomes of one-stage posterior surgery with bone grafting fusion and internal fixation, combined in half of cases with local continuous chemotherapy and postural drainage, for the treatment of lumbar vertebral tuberculosis. To our knowledge, this is the first study to compare the outcomes of these two treatment approaches. The results indicate that this one-stage surgery with local chemotherapy and postural drainage effectively eliminates the focal abscess and necrotic tissues, controls local inflammation, and accelerates graft bone fusion. Posterior instrumentation can accelerate interbody vertebral fusion, increasing spinal stability; it can also promote neurological recovery and accelerate treatment of tuberculosis [8, 17]. In the present study, patients with lumbar tuberculosis treated by posterior one-stage surgery showed various degrees of recovery from neurological deficits after surgery. However, interbody fusion time was significantly shorter in patients whose treatment involved continuous-irrigation chemotherapy and postural drainage than in patients who underwent the same surgery without these components. ESR and CRP levels were also significantly lower at 6 weeks postoperatively in the chemotherapy and postural drainage group than in the posterior approach–alone group. These findings indicate that local chemotherapy and postural drainage can enhance the reconstruction of segmental stability. After spinal tuberculosis lesions were eliminated, psoas abscesses could not be removed completely. At this point, the lesions and residual cavities were interlinked, and pus may have flowed into the bone lesions. Moreover, bleeding and exudation, often found after surgery, immersed the bone lesions in fluids, affecting recovery. Local rinsing, chemotherapy, and drainage can remove blood, exudation, and pus from the lesion in a timely manner; these procedures can also prevent fluid retention, reduce lesion pressure, and improve the local blood supply, thereby enhancing local tissue repair and promoting the healing of bone tissue. In addition, these procedures increase the concentration of anti-tuberculosis drugs in the lesion, inhibit the progression of pathological changes, and promote the limitation and healing of the lesion. The limitation of spinal tuberculosis lesions can create a microenvironment more closely approximating normal bone tissue, which is more conducive to bone repair.

A unilateral psoas abscess was observed in one patient in group A at 10 months postoperatively. No such outcome was observed in any patient in group B. Abscess remnants may be the result of large abscess size, preventing complete surgical elimination, and poor local blood supply, which reduces the local bactericidal capability of anti-tuberculosis drugs. Local continuous chemotherapy and postural drainage can solve those problems, clearing abscess remnants and necrotic tissue and thereby promoting lesion healing.

This study has several limitations. First, the sample was relatively small and insufficiently homogenous. In addition, lengthy intubation may cause sinus development, although this complication was not observed in any patient in either study group.

## Conclusions

One-stage posterior surgery involving debridement, bone grafting and internal fixation, and instrumentation with local continuous chemotherapy and postural drainage is an effective surgical treatment for lumbar spinal tuberculosis. This simple approach eliminated infection foci caused by abscess remnants and accelerated interbody fusion, resulting in a distinct curative effect with minor intraoperative trauma and few complications. We thus recommend this procedure for the treatment of lumbar spinal tuberculosis.

## References

[1] Lee TC, Lu K, Yang LC, Huang HY, Liang CL (1999). Transpedicular instrumentation as an adjunct in the treatment of thoracolumbar and lumbar spine tuberculosis with early stage bone destruction. J Neurosurg..

[2] Huang QS, Zheng C, Hu Y, Yin X, Xu H, Zhang G, Wang Q (2009). One-stage surgical management for children with spinal tuberculosis by anterior decompression and posterior instrumentation. Int Orthop..

[3] Jain AK, Dhammi IK, Prashad B, Sinha S, Mishra P (2008). Simultaneous anterior decompression and posterior instrumentation of the tuberculous spine using an anterolateral extrapleural approach. J Bone Joint Surg..

[4] Benli IT, Acaroglu E, Akalin S, Kis M, Duman E, Un A (2003). Anterior radical debridement and anterior instrumentation in tuberculosis spondylitis. Eur Spine J.

[5] Pang X, Wu P, Shen X, Li D, Luo C, Wang X (2013). One-stage posterior transforaminal lumbar debridement, 360°interbody fusion, and posterior instrumentation in treating lumbosacral spinal tuberculosis. Arch Orthop Trauma Surg.

[6] Mehta JS, Bhojraj SY (2001). Tuberculosis of the thoracic spine: a classification based on the selection of surgical strategies. J Bone Joint Surg (Br).

[7] Li J, Li XL, Zhou XG, Zhou J, Dong J (2014). Surgical treatment for spinal tuberculosis with bilateral paraspinal abscess or bilateral psoas abscess: one-stage surgery. J Spinal Disord Tech.

[8] Sun L, Song Y, Liu L, Gong Q, Zhou C (2013). One-stage posterior surgical treatment for lumbosacral tuberculosis with major vertebral body loss and kyphosis. Orthopedics.

[9] Ma YZ, Cui X, Li HW, Chen X, Cai XJ, Bai YB (2012). Outcomes of anterior and posterior instrumentation under different surgical procedures for treating thoracic and lumbar spinal tuberculosis in adults. Int Orthop.

[10] Buyukbebeci O, Seckiner I, Karsli B, Karakurum G, Başkonuş I, Bilge O (2012). Retroperitoneoscopic drainage of complicated psoas abscesses in patients with tuberculous lumbar spondylitis. Eur spine j off publ Eur Spine Soc Eur Spinal Deform Soc Eur Sect Cerv Spine Res Soc..

[11] Fairbank JC, Pynsent PB (2000). The Oswestry Disability Index. Spine (Phila Pa 1976).

[12] Kim KT, Lee SH, Lee YH, Bae SC, Suk KS (2006). Clinical outcomes of 3 fusion methods through the posterior approach in the lumbar spine. Spine..

[13] Hirakawa A, Miyamoto K, Takahiro M, Fukuta S, Hosoe H, Iinuma N (2010). Surgical outcome of 2-stage (posterior and anterior) surgical treatment using spinal instrumentation for tuberculous spondylitis. J Spinal Disord Tech..

[14] Fukuta S, Miyamoto K, Masuda T, Hosoe H, Kodama H, Nishimoto H, et al. Two-stage (posterior and anterior) surgical treatment using posterior spinal instrumentation. Spine. 2003;28:E302–8. 10.1097/01.BRS.0000083318.40123.5E 12897509

[15] Zhang HQ, Lin MZ, Shen KY, Ge L, Li JS, Tang MX, Wu JH, Liu JY (2012). Surgical management for multilevel noncontiguous thoracic spinal tuberculosis by single-stage posterior transforaminal thoracic debridement, limited decompression, interbody fusion, and posterior instrumentation (modified TTIF). Arch Orthop Trauma Surg.

[16] Guven O, Kumano K, Yalcin S, Karahan M, Tsuji S (1976). A single stage posterior approach and rigid fixation for preventing kyphosis in the treatment of spinal tuberculosis. Spine.

[17] Pang X, Shen X, Wu P, Luo C, Xu Z, Wang X (2013). Thoracolumbar spinal tuberculosis with psoas abscesses treated by one-stage posterior transforaminal lumbar debridement, interbody fusion, posterior instrumentation, and postural Drainage. Arch Orthop Trauma Surg.

